# P-1901. Clinical Tropical Medicine and Travelers’ Health for the Infectious Disease Provider - A Self-Paced Supplement for a Global Infectious Disease Education

**DOI:** 10.1093/ofid/ofaf695.2070

**Published:** 2026-01-11

**Authors:** Meredith Kavalier, Megan Shaughnessy, Matthew Enriquez, Beth Scudder, Kristina Krohn

**Affiliations:** University of Minnesota, Minneapolis, MN; Hennepin Healthcare System, Minneapolis, Minnesota; University of Minnesota, Minneapolis, MN; University of Minnesota, Minneapolis, MN; University of Minnesota, Minneapolis, MN

## Abstract

**Background:**

Five percent of the Infectious Disease board exam focuses on Tropical Medicine and Travelers’ Health and exposure to these topics in fellowship varies greatly. This often leaves Infectious Disease practitioners unprepared to manage global infections. The University of Minnesota started an asynchronous online course in 2015 focusing on Travel and Tropical Medicine specifically for Infectious Disease specialists to address this gap. The course is accredited for >35 CME hours and includes individual sections focusing on parasites, bacteria, viruses, fungi, and traveler’s health. Modules are available online for participants that currently practice in Infectious Diseases or are training to do so.

**Figure 1:**
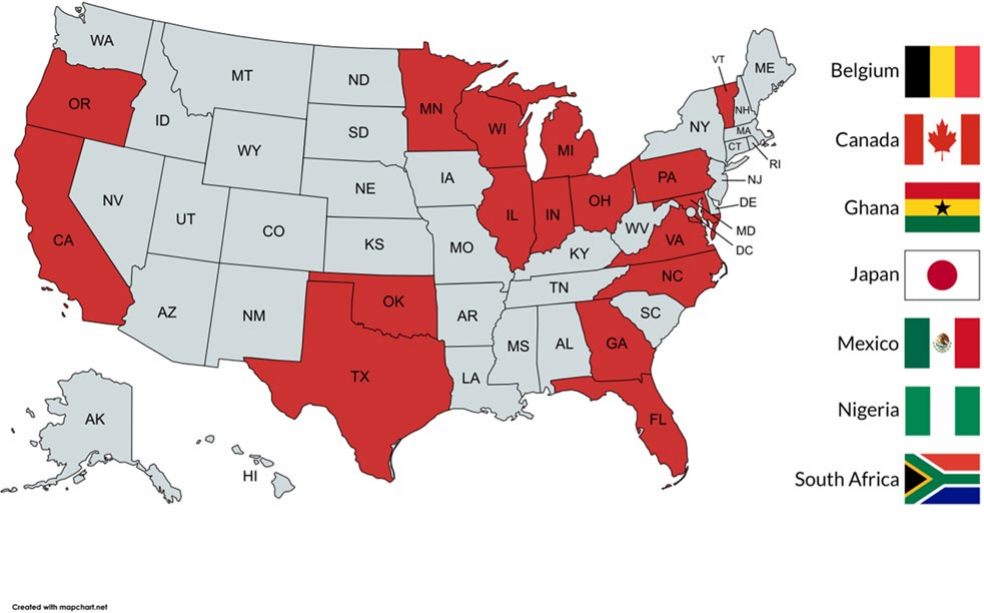
Map including states and countries participants reported residing in. States highlighted in red had at least one participant.

**Figure 2:**
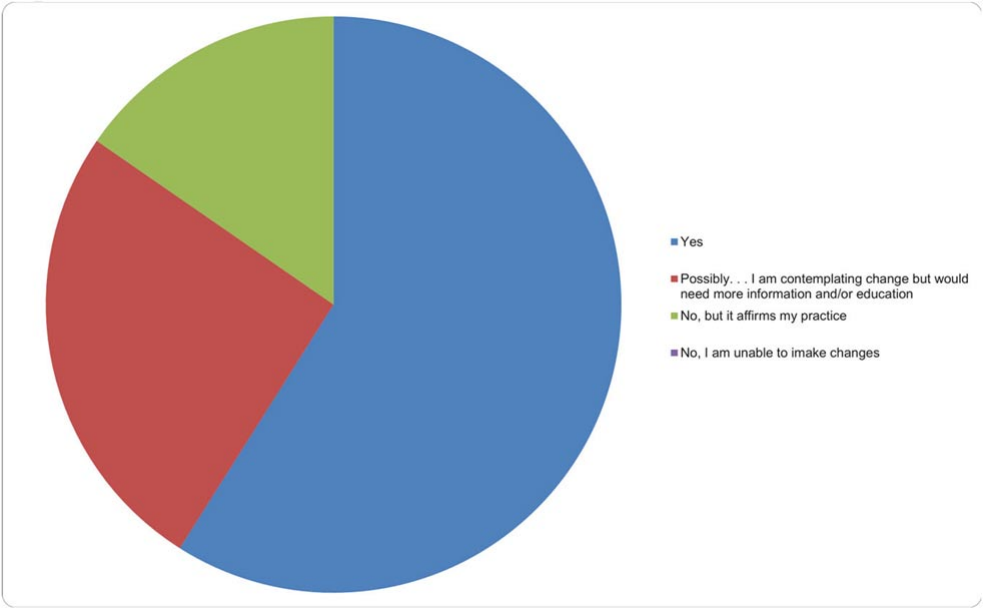
Graphical representation of responses to the question: “As a result of participating in this activity, will you make any measurable changes in your role?” Yes (23, 59.0%); Possibly. . . I am contemplating change (10; 25.6%); No, but it affirms my practice (6; 15.4%); No, I am unable to implement the suggested changes (0; 0%).

**Methods:**

This project involved the analysis of course assessments for the various sessions in the course, including pre/post knowledge, skills and attitude tools. Participant evaluations consisted of multiple choice questions, Likert scales, and short answer responses.

**Figure 3:**
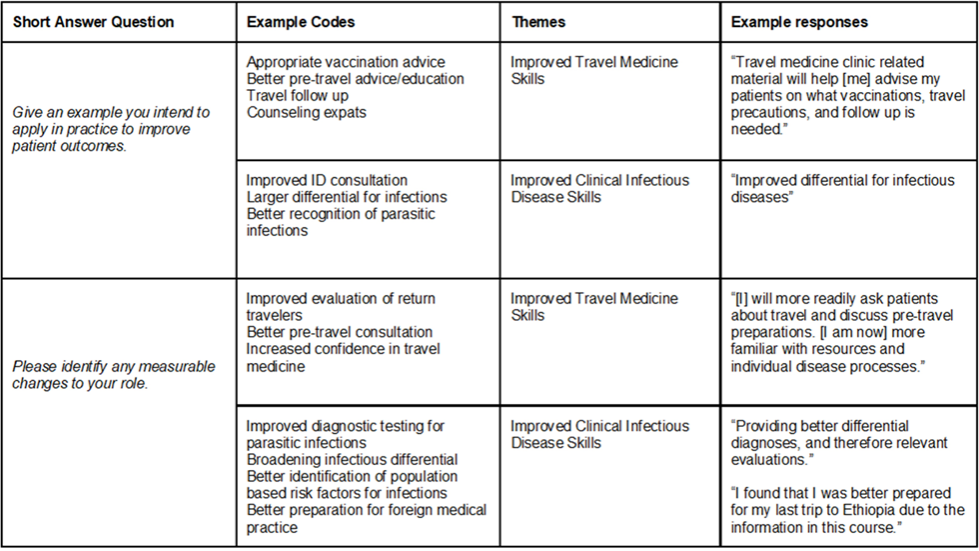
For those that answered “yes” to making changes in clinical practice, the short-answer examples provided were coded and thematically analyzed. The themes of “improved Travel Medicine skills” and “improved clinical Infectious Disease skills” were recurrent.

**Results:**

39 participants completed the course - 27 MDs, 9 advanced practice providers, 3 other. Registrants reported residency in 17 states and 7 countries (Fig. 1). Participants ranked individual sections highly, and 59% reported that they would make changes in their current practice based on course material (Fig. 2). Individuals indicating they would make a practice change provided short answer examples that were analyzed and thematically coded. The themes of improving infectious disease clinical care and improving travel advice were most frequently observed (Fig. 3).

**Conclusion:**

The University of Minnesota’s *Travel & Tropical Medicine for the Infectious Disease Specialis*t provides a unique and flexible supplemental education that is not currently covered by ID fellowships. Current trainees or practicing providers looking to expand their knowledge base and improve clinical care could consider enrollment.

**Disclosures:**

All Authors: No reported disclosures

